# Response surface modelling of Fenton pre-treatment of slaughterhouse sludge for enhanced anaerobic digestion

**DOI:** 10.1038/s41598-025-02731-3

**Published:** 2025-08-27

**Authors:** Mohsin Anwer, Mohd Ahmed Naim Shaikh, Saif Ullah Khan, Sayedali Mirkhalafi, Mohd Salim Mahtab, Izharul Haq Farooqi, Sohail Ayub, Mohammad Hadi Dehghani

**Affiliations:** 1 Department of Civil Engineering, Z. H. College of Engineering and Technology, Aligarh Muslim University, Aligarh, 202002 India; 2 Faculty of Engineering and Technology, School of Civil Engineering and Built Environment, Liverpool John Moores University (LJMU), Liverpool , UK; 3 Department of Environmental Health Engineering, School of Public Health, Tehran University of Medical Sciences, Tehran, Iran; 4 Center for Solid Waste Research, Institute for Environmental Research, Tehran University of Medical Sciences, Tehran, Iran

**Keywords:** Anaerobic digestion, Fenton process, Methanogenesis, Response surface methodology, Sludge conditioning, Environmental chemistry, Environmental impact, Chemical engineering, Engineering

## Abstract

Slaughterhouse sludge, a byproduct of meat processing, poses significant environmental risks if not properly treated, with potential impacts including water contamination and land pollution. Anaerobic digestion (AD) of this high-organic-content sludge offers a sustainable solution by facilitating biogas production, reducing reliance on fossil fuels, and enabling resource recovery. However, the complex nature of sludge necessitates pretreatment to enhance its biodegradability. In this study, the Fenton process, utilizing hydroxyl radicals (•OH) for oxidative breakdown of organic matter, was employed to improve the digestibility of slaughterhouse sludge. A response surface methodology (RSM)-based optimization approach, specifically the central composite design (CCD), was applied to investigate the effects of key operational parameters—pH, ferrous ion (Fe^2+^) dosage, and hydrogen peroxide (H_2_O_2_) dosage—on sludge disintegration. The response variables analyzed were soluble chemical oxygen demand (sCOD) and volatile suspended solids (VSS) reduction. The optimal conditions were identified as a Fe^2+^ dosage of 7.2 mg/g total solids (TS), a H_2_O_2_ dosage of 130.4 mg/g TS, and a pH of 3. Under these conditions, sCOD and VSS degradation increased by 37.5% and 40.5%, respectively, resulting in a 31% increase in methane yield over a 20-day AD period compared to untreated sludge. These findings demonstrate that Fenton pre-treatment enhances the biodegradability of slaughterhouse sludge, thereby improving the efficiency of AD and contributing to more sustainable waste management practices.

## Introduction

The slaughter industry is one of the largest industries with economic significance for every nation in the livestock sector. India is the fifth-largest meat exporter globally among the many nations producing meat^1^. According to the Ministry of Food Processing, the country has 3600 recognised slaughterhouses, 9 modern butcheries, and 171 meat processing plants, which slaughter over 121 million livestock like sheep, pigs, goats and poultry and 36.9 million buffaloes annually for local use as well as for export^2,3^. Slaughterhouses have serious problems for the environment in terms of soil, water, and land pollution. Each slaughtering wastes 15 L of water on average, which equates to 630 million gallons of water annually in India. Various cleaning processes like washing the blood of slaughtered animals, sterilization of the equipment, packaging etc., the type of animal slaughtered and the processing procedure result in enormous water consumption every day^4,5^. As a result, excessive discharge of organics from effluent streams of slaughterhouses becomes a peculiar problem^6,7^. The presence of pathogenic microorganisms, along with elevated concentrations ofnitrogen, phosphorus, chlorides, suspended solids, and colloidal substances, has attracted significant research attention toward the effective treatment of slaughterhouse wastewater. These contaminants substantially contribute to increased eutrophic pollution levels, posing serious risks to both human and animal health^8,9^.

The treatment of municipal and industrial wastewater predominantly relies on biological processes. Although these methods effectively reduce pollutants, they generate significant quantities of waste activated sludge (WAS) that require proper handling and disposal^10,11^. Handling and disposal account for 30–40% of capital costs and 50–60% of operational costs in wastewater treatment facilities^12^. The inherent challenges associated with WAS include its high organic content, odour, and microbial contamination, necessitating pre-treatment before final disposal. Activated sludge systems convert dissolved and suspended organic contaminants into biomass and gases, but producing substantial sludge volumes daily. Biological sludge stabilization methods, such as anaerobic digestion (AD), suffer from significant limitations, including prolonged retention periods (30–40 days) and low digestion efficiencies, with volatile suspended solids (VSS) reductions typically limited to 40–50%^13^. The presence of extracellular polymeric substances (EPS) in WAS significantly hampers sludge hydrolysis, the rate-limiting stage in AD, and contributes to the formation of gel-like structures that complicate dewatering^14,15^. Despite efforts to enhance dewaterability and reduce sludge volume through chemical, electrochemical, sonication, and thermal pre-treatments, these methods often exhibit inconsistent efficacy and high costs^16^.

Advanced oxidation processes (AOPs) have emerged as promising technologies for addressing the limitations of conventional sludge treatment methods. AOPs leverage the high oxidative potential of hydroxyl radicals (OH) to degrade organic contaminants non-selectively, bypassing the hydrolysis stage of anaerobic digestion^17^. Among AOPs, the Fenton reaction has garnered considerable attention due to its environmental benefits, ability to reduce sludge volume, and potential for improving sludge biodegradability^18^. The Fenton process employs iron (II) to catalyze hydrogen peroxide decomposition, generating hydroxyl radicals that disrupt sludge flocs, degrade bacterial cells, and solubilize organic matter^19^. Studies have demonstrated the efficacy of Fenton pre-treatment in enhancing sludge solubilization and methane production. For instance, Erden and Filibeli reported superior sludge solubilization with Fenton-treated thermophilic anaerobic digestion, achieving a 26.8% higher reduction in volatile solids compared to controls^20^. Two-stage digestion systems incorporating Fenton pre-treatment produced 1.3 times more methane than single-stage thermophilic systems^21^. Additionally, Pilli et al. (2016) highlighted a 3.1-fold increase in net energy and a 15% boost in methane generation with Fenton pre-treatment^22^. However, despite these promising outcomes, the optimization of operational parameters—including pH, Fe^2+^ and H_2_O_2_ dosage less explored, limiting the widespread adoption of the Fenton process in sludge management, particularly for complex waste streams like slaughterhouse wastewater. There is a pressing need to develop more sustainable methods that minimize chemical usage while maximizing sludge digestibility.

Given the complexities involved in pre-treatment processes, optimization is essential to maximize their efficiency and sustainability. Response Surface Methodology (RSM) and Central Composite Design (CCD) are powerful statistical tools for optimizing multi-variable systems. Siddiqui et al. (2023) also demonstrated the effectiveness of RSM in optimizing Fenton pre-treatment conditions for effluent treatment plant (ETP) sludge from slaughterhouses^19^. To address these gaps, the current study focuses on applying the Fenton process as a pre-treatment for slaughterhouse sludge, optimizing key operational parameters (pH, Fe^2+^ dosage, and H_2_O_2_ dosage) using the response surface methodology (RSM) tool in the Design-Expert software. The design of experiments (DOE) tool, is increasingly prominent in the environmental and water sectors. Traditional experimental design and optimization methods like One Factor at a Time (OFAT) have several significant issues, such as inadequate resource allocation and challenges in identifying interaction effects among the variables^23^. By investigating its effect on sludge disintegration and biodegradability, the study aimed to provide valuable insights into enhancing anaerobic digestion efficiency for slaughterhouse wastewater. The effects of individual Fenton process parameters were evaluated both independently and in combination, with a particular focus on their influence on soluble chemical oxygen demand (sCOD) and volatile suspended solids (VSS) in sludge. The study assessed not only the isolated impact of each parameter but also explored the interactive effects, providing a comprehensive understanding of how these factors contribute to the degradation and stabilization of sludge components. This study also emphasizes the need for further research on pathogen reduction and the environmental sustainability of sludge management systems.

## Materials and methods

### Materials and chemicals

Waste activated sludge (WAS) was collected from the return pipe of a secondary clarifier sludge hopper of the sewage treatment facility at Al-Hamd Agro Foods Products Pvt. Ltd, Aligarh. Before usage, the sludge samples were settled and kept at 4 °C in a cold chamber to reduce biological and chemical reactions. An hour oxidation period was taken into account when conducting the studies. The maximum storage period was one week. High-purity grade hydrogen peroxide (H_2_O_2_), sodium hydroxide (NaOH), sulfuric acid (99%), and ferrous sulphate (FeSO_4_), were purchased from Advent Chembio Pvt. Ltd. The characteristics of the raw samples are presented in Table 1. Standard methods were used to determine total solids, volatile solids, and dissolved solids (APHA 22nd Edition)^24^. The sCOD (soluble chemical oxygen demand) was examined using the standard methodology (5220 B-APHA, 2005)^25^. Membrane filters were used to collect and filter the supernatant. The filtrate was analyzed for sCOD. Chemical oxygen demand was determined by the closed reflux titrimetric method. pH was measured using the pH meter, while TDS was measured using the HQ30 d Portable Meter LBOD 10,101 probe.

**Table 1:** Characteristics of Raw sludge sample.

Parameter	Unit	Min–Max	Mean
pH	–	6.8–7.4	7.2
Total solids (TS)	mg/L	29,470‒33,210	31,280
Total dissolved solid (TDS)	mg/L	14,200–13,050	13,430
Volatile solids (VS)	mg/L	18,270‒25,178	24,359
Total suspended solids (TSS)	mg/L	17,300‒18,400	17,850
Volatile suspended solids (VSS)	mg/L	11,200‒12,600	11,900
sCOD	mg/L	1860‒2280	2120

### Experimental procedure and design

#### Fenton pre-treatment

A preliminary study of WAS was conducted for the Fenton process to determine the working ranges of operating factors followed by the optimization of the Fenton process. Response surface methodology (RSM) based on central composite design (CCD) was applied taking into account the interactive impacts of process variables on two chosen responses, namely the elimination of VSS and the concentration of sCOD. Pre-treatment was applied using an appropriate effective range. Design-Expert software was used for the pre-treatment to determine the optimum operating conditions by considering the interactive effects of different variables and responses in pre-treatment for enhancing sludge digestibility and disintegration. The main operational factors that were optimised using RSM-based CCD were the dosages of H_2_O_2_, Fe^2+^, and pH. The CCD/RSM is used for experimental design, to obtain the interactive effects of variables and for the optimization of the process. The details regarding the process is well reported in our previous studies^19,23^.

**Fig. 1:**
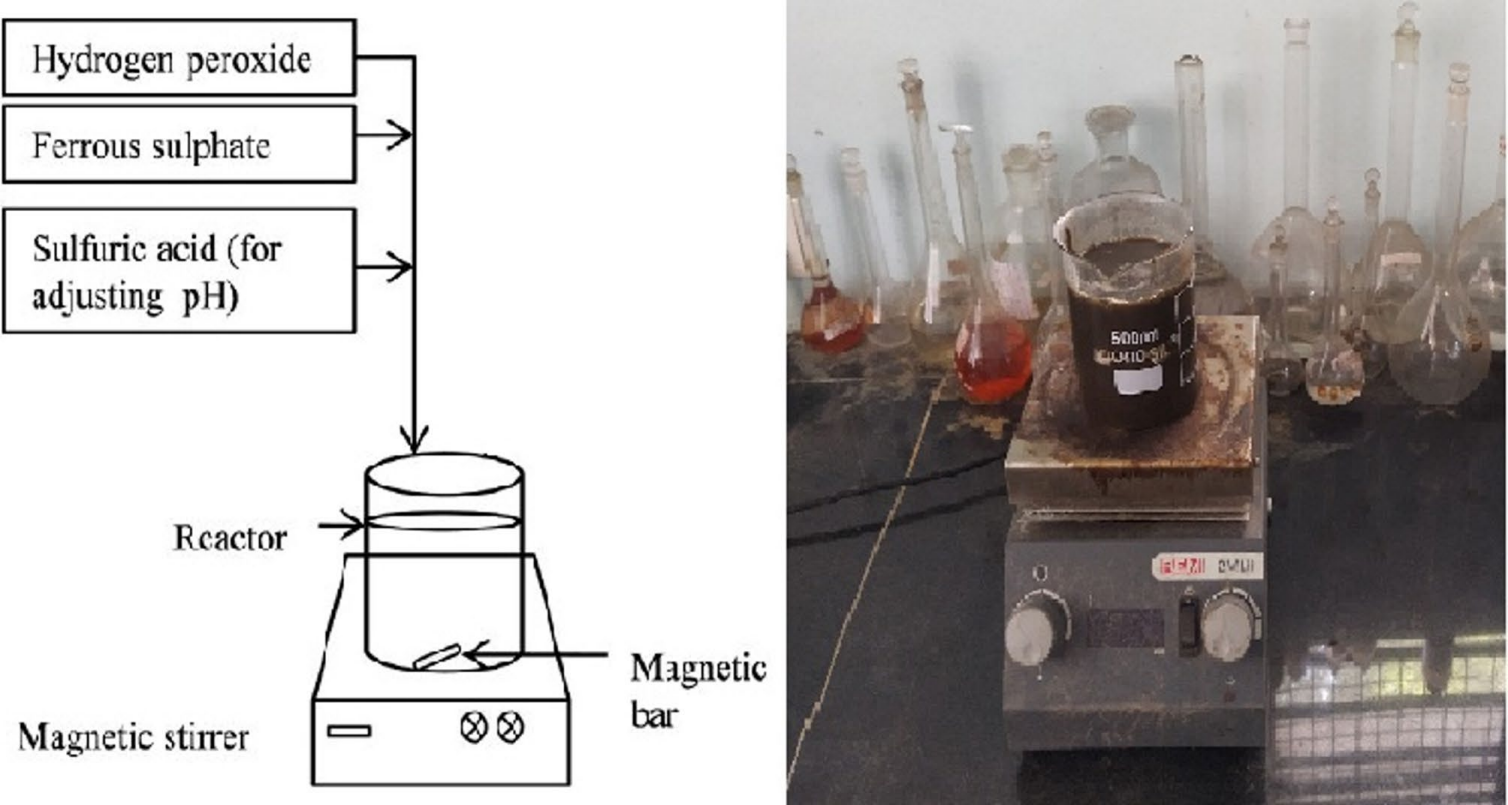
Experimental set-up for the Fenton process.

A total of 42 experimental runs were carried out on the experimental set-up for the Fenton process as shown in Fig. 1. To find the ideal sludge pre-treatment operating conditions, empirical second-order polynomial models were fitted with data. Table 2 shows the coded values of process parameters and their ranges. The Fenton process coded values for pH(A), mg H_2_O_2_/g TS (B) and mg (Fe^2+^) g TS (C) were set at 3 levels as per the design chart obtained by RSM as shown in the Table 2. The sludge sample of 0.5 L was taken for Fenton pre-treatment. By using H_2_SO_4_ and NaOH, the pH of the sludge was first adjusted to carry out the process. The Fe^2+^ was added in specific quantities in the second stage. After that, the sample received additions of various H_2_O_2_ concentrations. For 60 min, the combined sample was agitated at 100–500 rpm. Ferrous (FeSO_4_.7H_2_O), the source of Fe^2+^ in the Fenton tests, was of analytical grade. H_2_SO_4_ (98–99%) and a stable hydrogen peroxide solution (30% w/w) were both used. Effects of individual Fenton process parameters were evaluated individually as well as their interactive effects on the sCOD and VSS of sludge. Completely mixed anaerobic digestion was carried out in two similar batch digesters on the pretreated and untreated WAS to assess each digester’s efficiency in terms of biogas output.

**Table 2:** Coded values of process parameters and their ranges.

Factor	Name	Units	Minimum	Maximum	Coded Low	Coded High	Mean
A	pH		2.50	4.50	− 1 ↔ 2.50	+ 1 ↔ 4.50	3.49
B	H_2_O_2_	mg/g TS	10.00	200.00	− 1 ↔ 10.00	+ 1 ↔ 200.00	96.67
C	Fe^2+^	mg/g TS	2.00	10.00	− 1 ↔ 2.00	+ 1 ↔ 10.00	5.90

### Biochemical methane potential (BMP)

Based on the BMP assay, the impact of Fenton pre-treatment on the anaerobic biodegradability of slaughterhouse sludge was assessed. For comparison, the BMP test was run on both untreated (R) and treated (S) samples. In 1000 mL bottles with a 300 mL reaction volume, BMP tests were conducted. To create anaerobic conditions, all bottles were purged with a gas combination that was 75% N_2_ and 25% CO_2_ for 3–4 min. To prevent gas leaking from the bottles, rubber stoppers and screw caps were used. The bottles were placed in a water bath that was kept at a constant 37 °C. Daily biogas gas productions were monitored using the liquid displacement method with distilled water and 3% NaOH (w/v)^26^. Biogas production in the bottles served as a gauge for the sludge anaerobic digesting performance. High anaerobic digestion performance of the sludge samples was indicated by high biogas outputs in the bottles.

### Anaerobic digestion of raw and pre-treated WAS

The 20-day monitoring of cumulative methane production in serum bottles reveals that pre-treating sludge may be a viable option for enhancing anaerobic degradation. Only 600 mL of methane was produced by the raw sludge, whereas 785 mL was collected after the 20-day incubation period for the treated sludge with the recommended dosage. In this case, the Fenton-treated sludge produced over 31% more methane than the untreated sludge. The initial biodegradability of the sludge determines the rise in methane generation, with greater effects on hardly biodegradable sludge, and has been correlated linearly with sludge COD solubilization. The improvement in biogas generation from pre-treated sludge demonstrated that pre-treatment could lessen the impact of the rate-limiting phase. Due to pre-treatment WAS hydrolysing a large amount of organic waste into soluble forms, it was used right awayin the anaerobic digestion process. Pre-treatment could cause the release of organic matter from inner to outer portions, speeding up the hydrolysis of granular organics and increasing digestive effectiveness.

## Results and discussion

### Analysis of variance (ANOVA)

ANOVA was used to evaluate the “goodness of fit” of the data. The F-test at the 5% level of confidence found that the models for VSS removal and sCOD were significant. The fitted regression model was developed to describe the effects of factors on pre-treatment. If the p-values are less than 0.05, the relevant factors significantly affect the responses^27^. The two pre-treatment responses are statistically significant in the models (*p* < 0.05). Higher p-valued interaction terms have little to no effect on the model^23^. It is clear that the goodness-of-fit value for the regression models, R^2^, 0.9944, is suitable. A high R^2^ value confirms that the obtained model adequately described the pre-treatment process. Table 3 shows the analysis of variance for the response variable sCOD. Table 4 shows the fit statistics for COD with an R^2^ value of 0.9944 which indicates a good model. The predicted R^2^ of 0.9418 and the adjusted R^2^ of 0.9928 are reasonably in agreement; that is, the difference is less than 0.2. The Lack of Fit is not significant which is favorable to the model. Furthermore, the Adeq Precision value is greater than the desired value (A.P. > 4) which further confirms the obtained quadratic model is significant^23^. Similarly, Table 5 shows the analysis of variance for the response variable VSS. The Model F-value of 1161.93 suggests that the model is significant. When the P-value is less than 0.0500, model terms are deemed significant^27^. The lack of fit is not significant. The discrepancy between the Predicted R^2^ of 0.9875 and the Adjusted R^2^ of 0.9961 is less than 0.2, which is considered to be a reasonable agreement as evident from Table 6.

#### Response 1: ANOVA for sCOD

**The final equation of sCOD in terms of actual factors**

**sCOD =** 2192.08 + −202.656 * pH + 13.4754 * H2O2 + 83.5287 * Fe2 + + 0.100821 * pH * H2O2 + 2.4737 * pH * Fe2 + + 0.0359853 * H2O2 * Fe2 + + 13.2735 * pH^2 + −0.0544267 * H2O2^2 + −6.71644 * Fe2+^2.

**Table 3:** ANOVA for quadratic model.

Source	Sum of Squares	df	Mean Square	F-value	*p*-value	
**Model**	3.775E + 06	9	4.194E + 05	632.37	< 0.0001	significant
A-pH	2.648E + 05	1	2.648E + 05	399.28	< 0.0001	
B- H_2_O_2_	2.293E + 06	1	2.293E + 06	3457.45	< 0.0001	
C-Fe^2+^	1.458E + 05	1	1.458E + 05	219.83	< 0.0001	
AB	3362.18	1	3362.18	5.07	0.0314	
AC	3607.38	1	3607.38	5.44	0.0261	
BC	6844.84	1	6844.84	10.32	0.0030	
A²	274.25	1	274.25	0.4135	0.5248	
B²	3.741E + 05	1	3.741E + 05	564.08	< 0.0001	
C²	22670.99	1	22670.99	34.18	< 0.0001	
**Residual**	21224.59	32	663.27			
Lack of Fit	21008.24	3	7002.75	938.66	< 0.0001	Not significant
Pure Error	216.35	29	7.46			
**Cor Total**	3.796E + 06	41				

**Table 4:** Fit statistics.

Std. Dev.	25.75	R^2^	0.9944
Mean	2273.83	Adjusted R^2^	0.9928
C.V. %	1.13	Predicted R^2^	0.9418
		Adeq Precision	82.4412

#### Response 2: ANOVA for VSS

**The final equation in terms of actual factors**

VSS = 9710.11 + 378.03 * pH + −39.0643 * H2O2 + −261.427 * Fe2+ + −1.2252 * pH * H2O2 + −6.08223 * pH * Fe2+ + −0.116162 * H2O2 * Fe2 + + 11.8748 * pH^2 + 0.163414 * H2O2^2 + 20.0171 * Fe2+^2.

**Table 5:** ANOVA for quadratic model.

Source	Sum of Squares	df	Mean Square	F-value	*p*-value	
**Model**	4.774E + 07	9	5.304E + 06	1161.93	< 0.0001	significant
A-pH	3.264E + 06	1	3.264E + 06	715.13	< 0.0001	
B-H2O2	3.177E + 07	1	3.177E + 07	6960.72	< 0.0001	
C- Fe^2+^	1.848E + 06	1	1.848E + 06	404.76	< 0.0001	
AB	4.965E + 05	1	4.965E + 05	108.77	< 0.0001	
AC	21808.43	1	21808.43	4.78	0.0363	
BC	71324.39	1	71324.39	15.62	0.0004	
A²	219.50	1	219.50	0.0481	0.8278	
B²	3.373E + 06	1	3.373E + 06	738.86	< 0.0001	
C²	2.014E + 05	1	2.014E + 05	44.11	< 0.0001	
**Residual**	1.461E + 05	32	4564.83			
Lack of Fit	1.452E + 05	3	48396.55	1585.88	< 0.0001	Not significant
Pure Error	885.00	29	30.52			
**Cor Total**	4.788E + 07	41				

**Table 6:** Fit statistics.

Std. Dev.	67.56	R^2^	0.9969
Mean	9117.02	Adjusted R^2^	0.9961
C.V. %	0.7411	Predicted R^2^	0.9875
		Adeq Precision	108.2119

### Effect of process variables

**Fig. 2:**
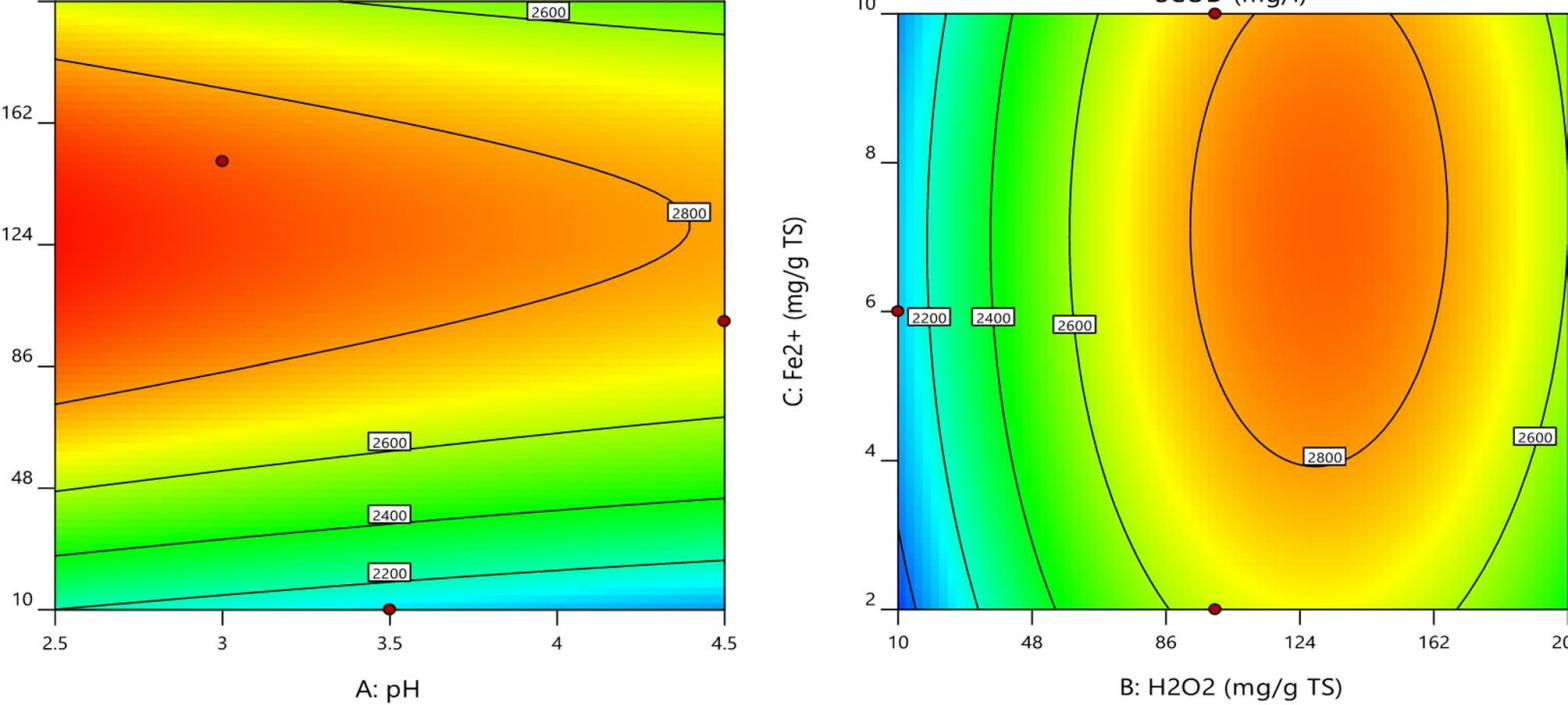
Contour plots between (**a**) H_2_O_2_ dosage and pH; (**b**) H_2_O_2_ dosage and Fe^2+^ dosage.

It was observed that with increasing H_2_O_2_ dosage at lower pH values, the sCOD concentration increased up to a certain extent as indicated by the red zone of the contour plot shown in Fig. 2a. This is due to more hydrolysis caused by •OH, whereas the further increase of dosage reduced the sCOD level because a higher amount of generated •OH caused complete mineralization of organic matter released from the cells of WAS. However, it is clear from the contour plot shown in Fig. 2b that initially increasing the values of both factors resulted in higher sCOD, however later on further increase caused reduction of sCOD values. This can be due to the inhibitory effect of hydroxyl radicals, which is found in correlation with past research works^28,29^. The 3D surface plots (Fig. 4) show that as the pH values were raised the sCOD levels decreased. The main cause of this reduction was the decreased amount of free Fe^3+^ brought on by the production of Fe (OH)_3_ molecules, which has a slow reaction rate with H_2_O_2_ during the process. Additionally, as the sludge pH increased, the production of oxidized free •OH radicals decreased. However, the impact of acidification on the disintegration of sludge was only marginal^30^. Because of this, the ideal pH for the greatest sludge separation with the least amount of chemical consumption to adjust the sludge pH was discovered to be 3, even though the maximum sCOD concentration was attained at pH 2.5^31^. It has been seen that sCOD concentration increased when H_2_O_2_ was delivered in low dosages but decreased when H_2_O_2_ dosage was increased. The hydroxyl radicals in the process damaged organic compounds and microorganisms in the biomass by oxidizing cell walls and dissolving organic matter at doses less than 130 g H_2_O_2_/kg TS. The amount of sCOD in the liquid phase increased as a result of the dissolved organic matter being released into it. However, a decrease in the concentration of sCOD dosages above 130 H_2_O_2_/kg TS in the liquid phase can be explained by the presence of •OH radicals, which have a high oxidation potential and are capable of converting organic matter into water and carbon dioxide while also preventing the disintegration of sludge. Previous studies have also demonstrated that hydroxyl radicals have an inhibiting effect^28^.

**Fig. 3:**
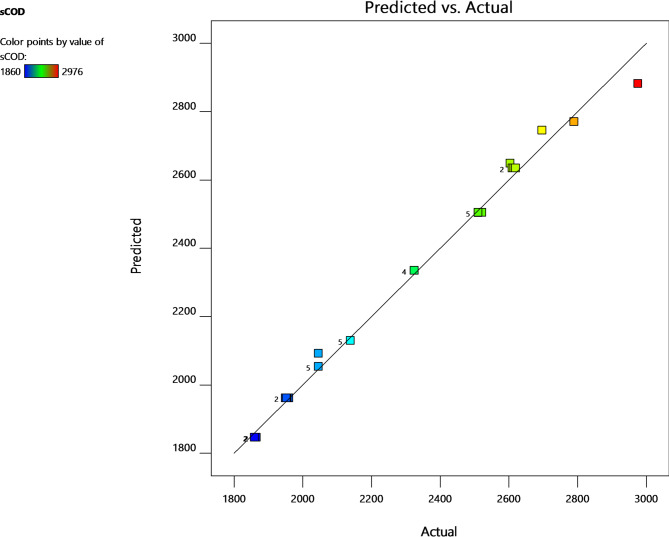
Actual versus predicted response for sCOD.

In the Fenton reaction, the Fe^2+^ ion immediately interacts with H_2_O_2_ to produce •OH. As the concentration of iron ions increases, so does the rate of disintegration. Over a certain concentration, the rate of disintegration remains quite low. The sCOD value increases as the iron concentration increases up to a certain level. However, further increases in iron levels create a negative impact on •OH radical generation, which resulted in a significant declination in sCOD values. The iron dosages were discovered to be 7.2 mg/g TS after taking into account these findings. A diagnostic plot (Fig. 3) comparing the anticipated versus actual values can be used to evaluate the model’s effectiveness. The idealised trend’s linear distribution of the points shows that the anticipated values are fairly close to the corresponding observed values. This is also confirmed by the significant model term with a p-value less than 0.0001.

**Fig. 4:**
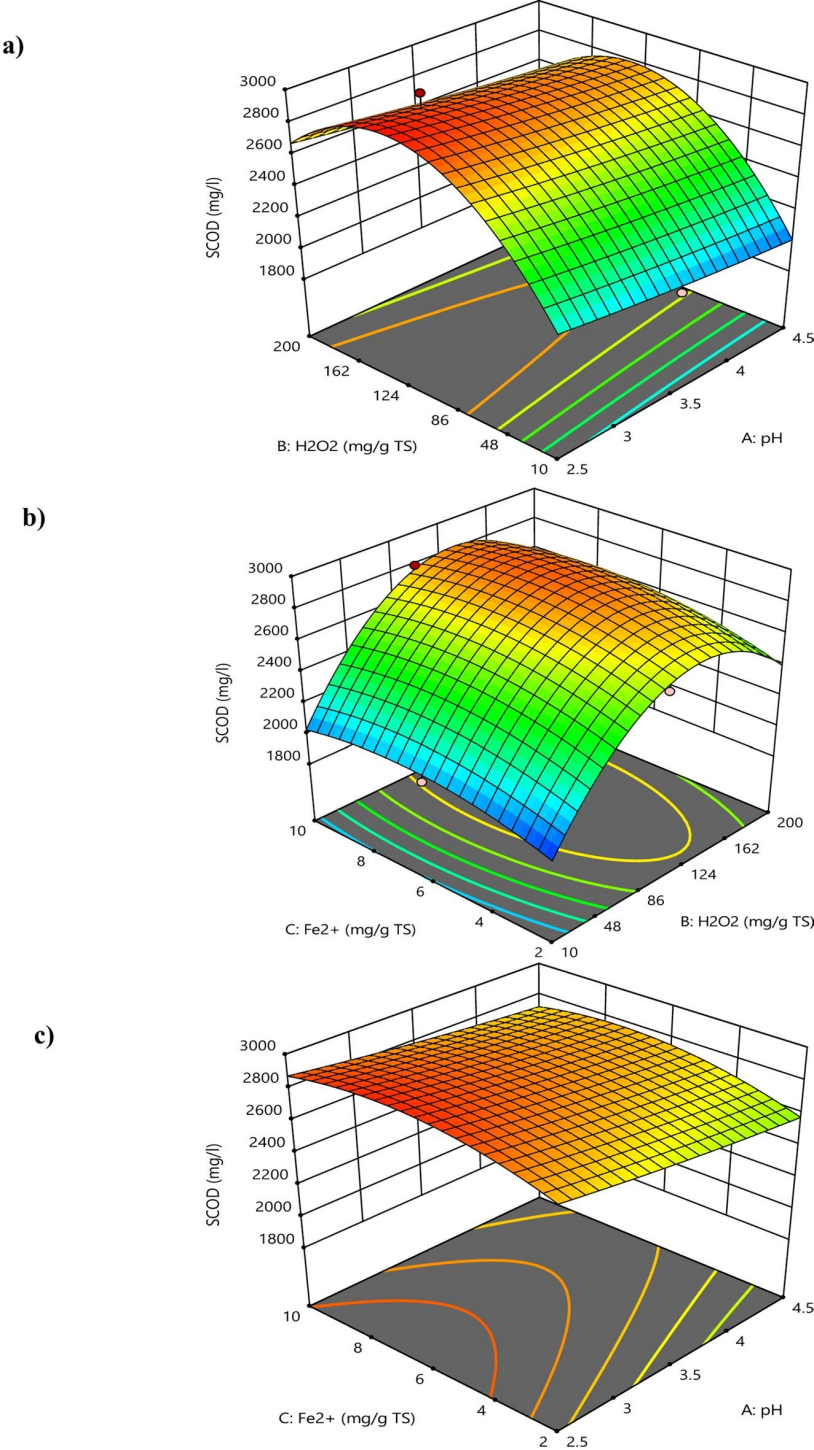
Response surface plots of sCOD, as a function of dosage of (**a**) mg H_2_O_2_/g TS and pH, (**b**) pH and mg Fe^2+^/g TS, (**c**) mg H_2_O_2_/g and mg Fe^2+^/g TS.

As can be seen in 3D surface plots in Fig. 4, there was an initial decrease followed by an increase in sCOD concentration as the H_2_O_2_ dosage was increased. The cytoplasm that was eluted from the decomposing microbe was degraded, and the surplus sludge was then solubilized by hydroxyl radicals, which caused the decrease in sCOD. Cell lysis contributed to the organic loading and the increase in sCOD concentration by releasing cell contents into the sludge slurry^32^. Additionally, excessive amount of H_2_O_2_ can lead to its auto-decomposition into water and oxygen (Eq. 1) and the recombination of •OH, which lowers the concentration of •OH and decreases the efficiency of degradation. Also due to the mineralization of released organic material at higher doses overall efficiency decreases.

1 $$2H_2O_2 \longrightarrow 2H_2O+O_2$$

**Fig. 5:**
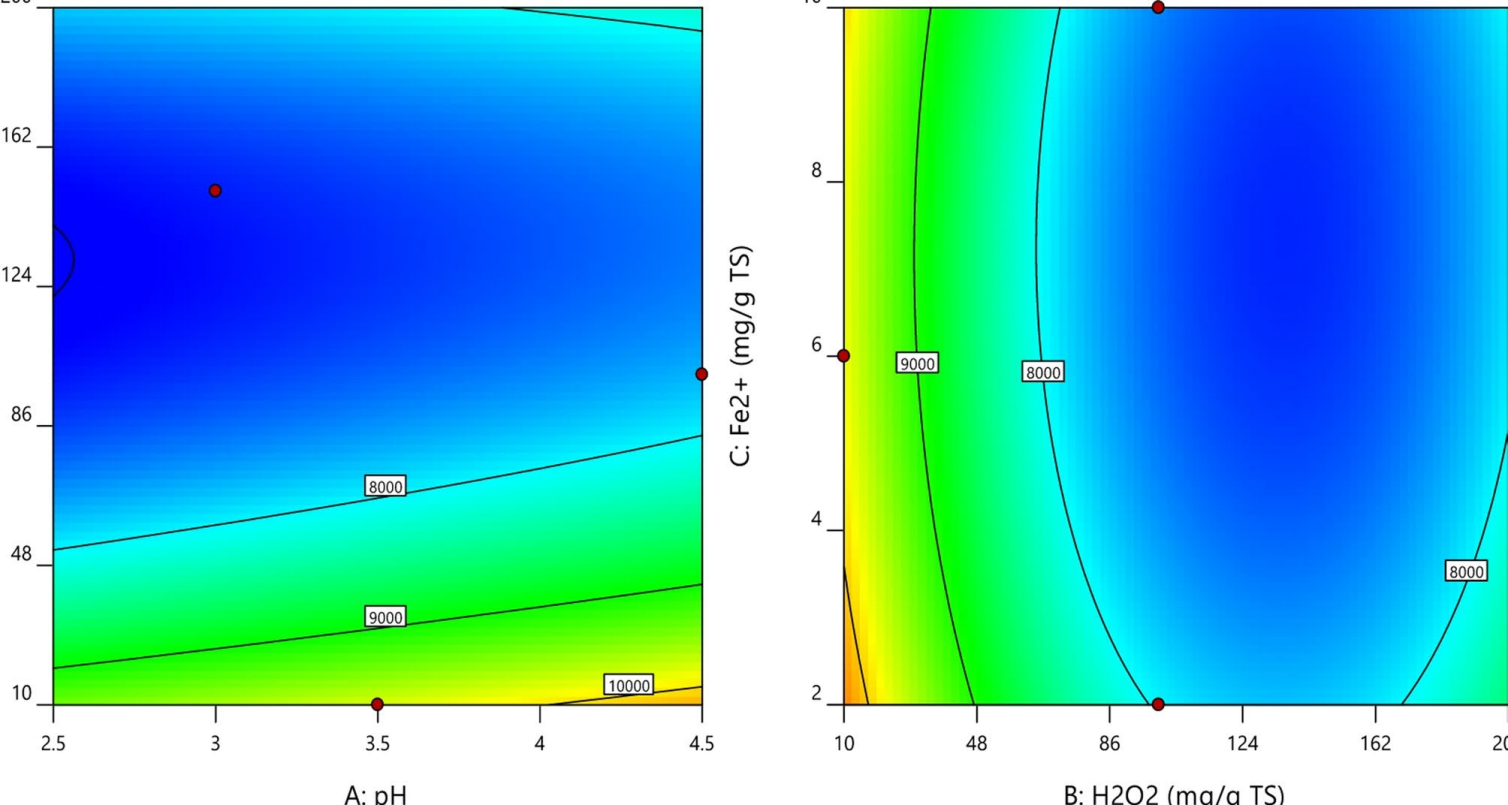
Contour plots (**a**) between H_2_O_2_ dosage and pH; (**b**) between H_2_O_2_ dosage and Fe^2+^ dosage.

**Fig. 6:**
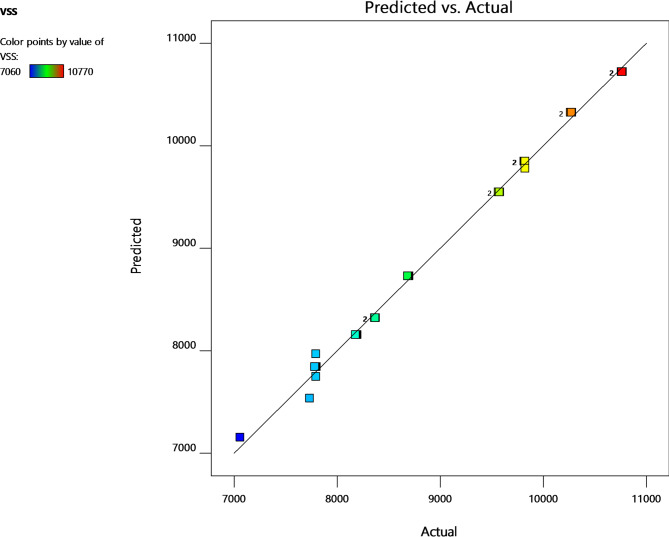
Actual versus predicted response for VSS.

**Fig. 7:**
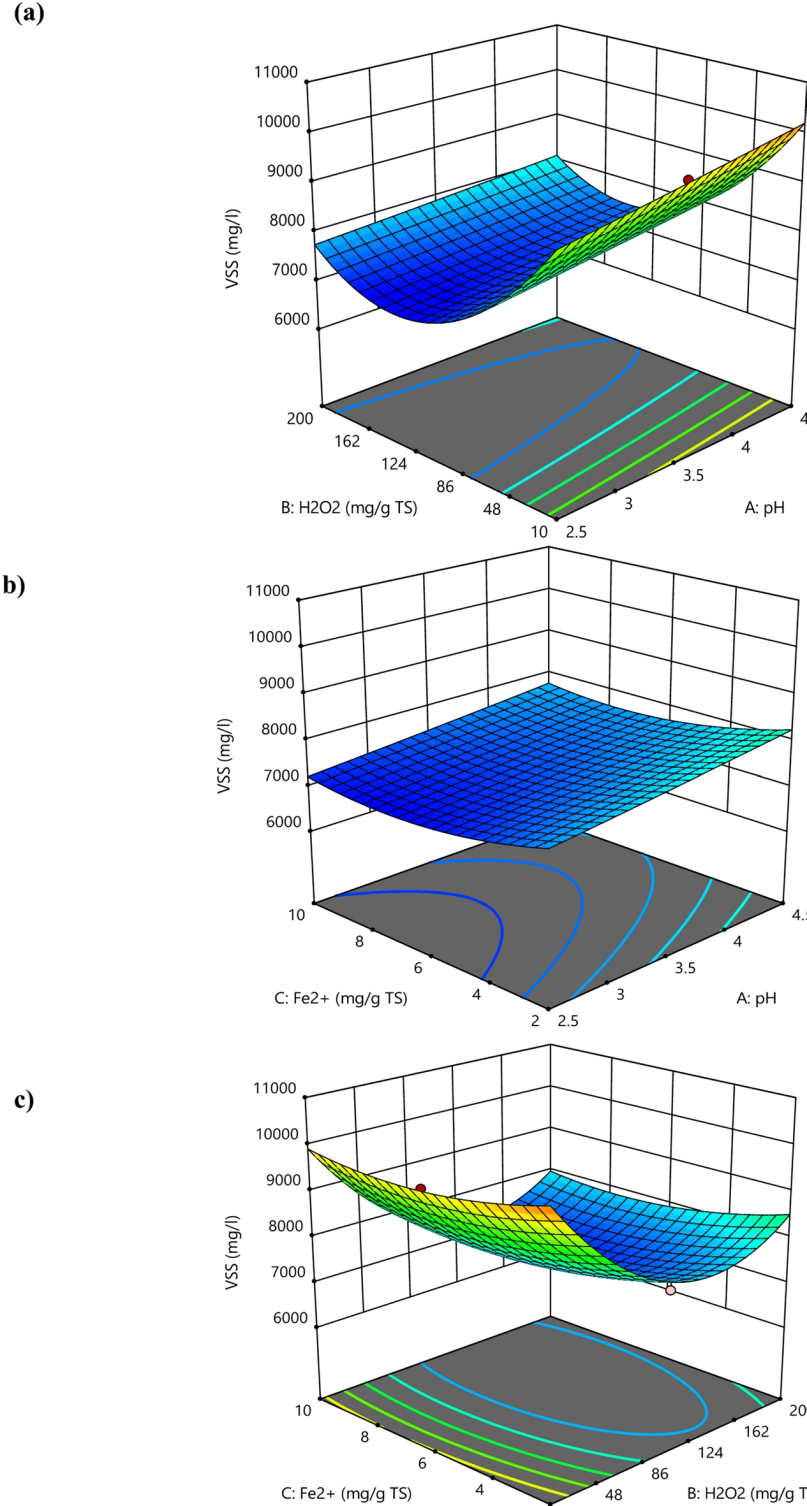
Response surface plots of VSS, as a function of dosage of (**a**) mg H_2_O_2_/g TS and pH, (**b**) pH and mg Fe^2+^/g TS, (**c**) mg H_2_O_2_/g and mg Fe^2+^/g TS.

Due to its ability to catalytically break down H_2_O_2_ and produce •OH, Fe^2+^ is a crucial part of the Fenton process. Because initial solubilization depends on Fe^2+^ concentration, raising the Fe^2+^ dosage raises sCOD concentration. However, if there is an excess of Fe^2+^ it starts to compete with organic molecules for the •OH, which reduces efficiency^22^. At increased Fe^2+^ dose, the coagulation process is also started, generating more sludge and raising the effluent’s total dissolved solids (TDS). The initial pH, which affects iron solubility, complexation, and redox cycling between states (II) and (III), limits the influence of Fe^2+^ dosage on the treatment. Because Fe^2+^ functions as a catalyst to speed up the conversion of H_2_O_2_ into •OH, its viability therefore depends on the pH of the solution. A lower concentration of Fe^2+^ has the opposite effect, decreasing the efficiency of solubilisation^33^. The concentration of sCOD increased somewhat when the initial pH was increased from 2.5 to 4.0, but it decreased significantly when the initial pH was elevated above 4.0. Two causes are identified as the root of the problem: first, lower pH values with less •OH restrict the effectiveness of Fenton oxidation, and second, higher pH values with more •OH reduce the activity of the Fenton reagent^34^. It was observed from the contour plots shown in Fig. 5a, that increasing H_2_O_2_ dosage at lower pH values, caused more degradation of VSS content up to a certain limit (Blue zone of contour plot), due to more oxidation and hydrolysis of WAS floc. However, a further increase in dosage reduces the VSS degradation levels. Also, at higher pH values the efficiency of the Fenton reagent decreases resulting in lesser VSS degradation as shown in 3D plots (see Fig. 7). Similar results were found in past study^34,35^. Furthermore, as the dosage of both H_2_O_2_ and Fe^2+^ increases, the degradation of VSS content increases as shown in Fig. 5b. However, after reaching at highest degradation, VSS content starts increasing with a further increase of both factors. This might be due to inhibition of sludge disintegration. Here, the actual vs. predicted plot from Fenton oxidation for VSS (Fig. 6) shows that the points were all well distributed and close to the fitted line. It was observed that there is no difference between the actual response and the predicted values by the model. This is also confirmed by the significant model term in Table 5 with a p-value less than 0.0001 (Fig. 7).

### Process optimization

**Fig. 8:**
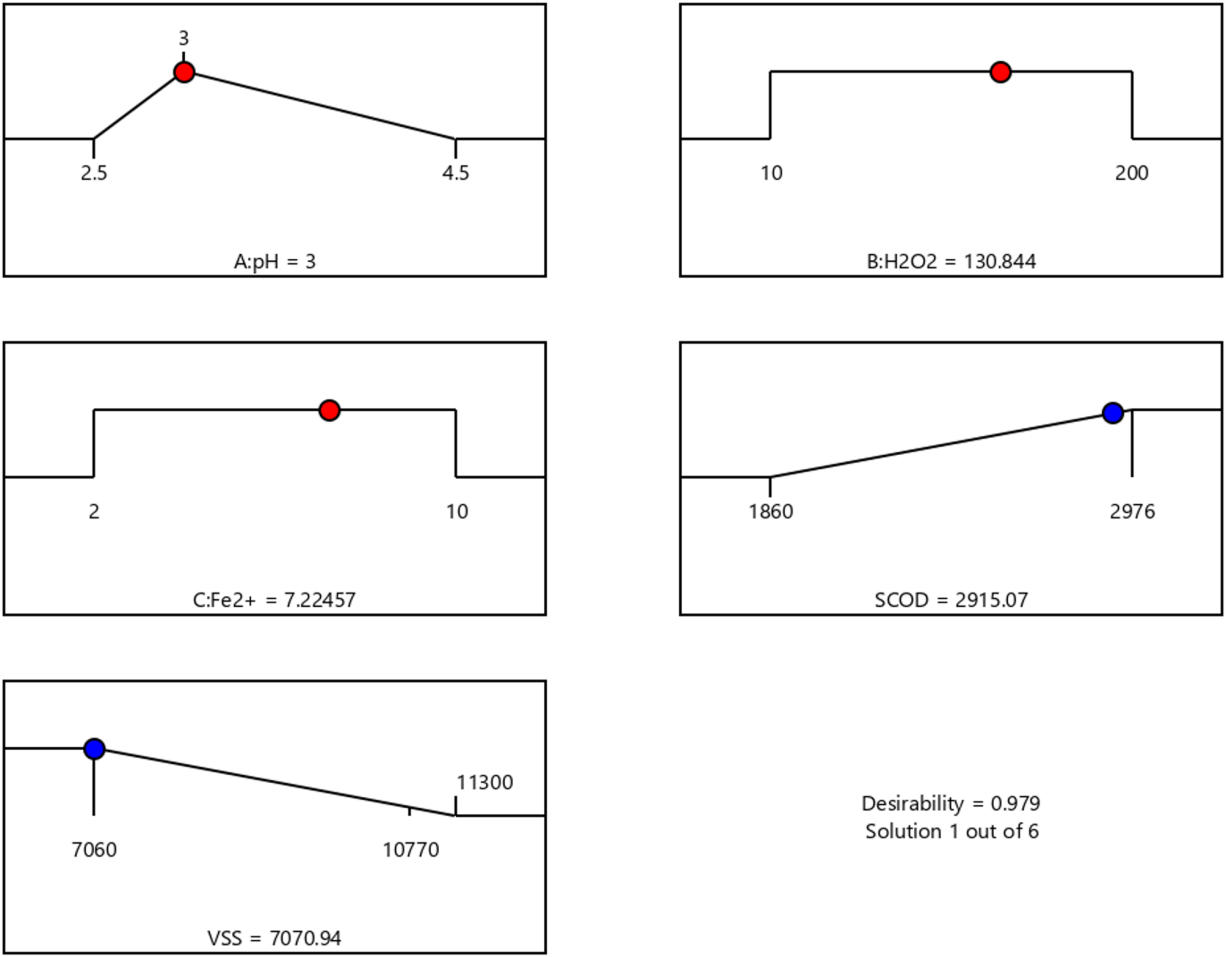
Values of variables and responses after optimization.

When there are numerous responses, it is possible to graphically depict the ideal operating conditions where all parameters concurrently match the desired response criteria^36^. The regions that meet the optimization requirements are shaded in the graphic representation of the area of a viable response value in the factor space. Response limitations were chosen as the least allowable values for each parameter near their achieved efficiency to obtain a relatively precise optimum zone – VSS elimination 40.5% and sCOD 2915 mg/L for Fenton oxidation. The values of the variables and responses following optimization are shown in Fig. 8.

## Conclusion

The present study was performed to enhance the anaerobic digestion of slaughterhouse sludge. Fenton pre-treatment was employed to improve the solubilization and disintegration of waste activated sludge. By using CCD/RSM, the role of process variables with the overall impact of Fenton pre-treatment on waste activated sludge (WAS) was optimized in terms of VSS removal and sCOD responses. Based on optimization and result analysis, 130.4 mg H_2_O_2_/g TS and 7.2 mg Fe^2+^/g TS dosage at pH 3 were found to be the best suitable operating conditions showing 31% enhancement of methane production as compared to raw WAS. However, due to chemical & energy consumption, the Fenton process alone may not be a sustainable method of sludge treatment due to which combination of methods, such as electrochemical peroxidation and electro-Fenton, are also employed so as to overcome these drawbacks along with increasing solubilization and disintegration and targeting higher recovery of methane. Furthermore, the generation of Fenton sludge which is one of the drawbacks of the process must be controlled and considered in future studies. To quantify and characterise the generated sludge in order to evaluate the overall process performance.

## Data Availability

The datasets generated and/or analyzed will be available from the corresponding author onreasonable request.
